# Frequent *CALR* exon 9 alterations in *JAK2* V617F-mutated essential thrombocythemia detected by high-resolution melting analysis

**DOI:** 10.1038/bcj.2015.21

**Published:** 2015-03-20

**Authors:** K-H Lim, Y-C Chang, C Gon-Shen Chen, H-C Lin, W-T Wang, Y-H Chiang, H-I Cheng, N-W Su, J Lin, Y-F Chang, M-C Chang, R-K Hsieh, Y-Y Kuo, W-C Chou

**Affiliations:** 1 Graduate Institute of Oncology, National Taiwan University College of Medicine, Taipei, Taiwan; 2 Division of Hematology and Oncology, Department of Internal Medicine, Mackay Memorial Hospital, Taipei, Taiwan; 3 Laboratory of Good Clinical Research Center, Department of Medical Research, Mackay Memorial Hospital, Tamsui District, New Taipei City, Taiwan; 4 Department of Medicine, Mackay Medical College, New Taipei City, Taiwan; 5 Institute of Molecular and Cellular Biology, National Tsing-Hua University, Hsinchu, Taiwan; 6 Division of Hematology and Oncology, Department of Internal Medicine, Mackay Memorial Hospital, Hsinchu, Taiwan; 7 Division of Hematology, Department of Internal Medicine, National Taiwan University Hospital, College of Medicine, National Taiwan University, Taipei, Taiwan; 8 Department of Laboratory Medicine, National Taiwan University Hospital, Taipei, Taiwan

Essential thrombocythemia (ET) is a clonal hematopoietic stem cell neoplasm and one of the classic *BCL-ABL1*-negative chronic myeloproliferative neoplasm (MPN), which also includes polycythemia vera and primary myelofibrosis (PMF).^1^ Recently, two seminal studies discovered a high frequency of somatic calreticulin (*CALR*) mutations in patients with *JAK2*/*MPL*-unmutated ET and PMF.^2, 3^ The pattern of most *CALR* mutations in MPN is heterozygous indels in exon 9 causing one-base pair (bp) reading frameshift. *CALR* mutations have been shown to have important diagnostic and prognostic significance in ET and PMF patients,^2, 3, 4^ and will likely be incorporated into the World Health Organization (WHO) diagnostic criteria for MPN. *In vitro* studies on the molecular pathogenesis of *CALR* mutations in MPN have shown controversial results in regard to the involvement and/or activation of the JAK/STAT signaling pathway,^2, 3^ and the exact pathogenesis of *CALR* mutations is not yet completely understood at the present time.^5^

Several techniques such as Sanger sequencing and polymerase chain reaction (PCR) followed by fragment analysis have been used to detect *CALR* mutations.^2, 3, 6, 7^ High-resolution melting analysis (HRMA) is a well-established method for the screening of mutations, and we have developed a rapid and sensitive HRMA for the detection of *CALR* exon 9 mutations.^8^ In this study, we sought to screen a cohort of 92 Taiwanese ET patients for *CALR* exon 9 mutations with HRMA and Sanger sequencing independently, and to determine the clinical and molecular correlates.

The institutional review board of Mackay Memorial Hospital has approved the screening for mutations. All patients provided written informed consent. Diagnosis of ET was established on the basis of the 2008 WHO criteria. The clinical and laboratory characteristics at the time of diagnosis or referral were collected. Genomic DNAs derived from the bone marrow, peripheral blood and peripheral blood granulocytes and/or mononuclear cells were used for mutation screening. *CALR* mutations were screened by Sanger sequencing on an ABI 3730 sequencer as preciously described.^3^ *CALR* exon 9 mutations were independently screened by HRMA using a CFX96 real-time PCR detection system (Bio-Rad Laboratories, Hercules, CA, USA) as previously described with a maximal sensitivity of 2.5% for both *CALR* type 1 and type 2 mutants.^8^ Briefly, a pair of oligonucleotide primers were used to amplify a 134-bp amplicon (GenBank: NM_004343), which flanked all *CALR* exon 9 variants reported in MPN. All samples with distinguished melting curves from wild type were confirmed by duplicate studies. Peripheral blood samples from 78 healthy adults were also used to validate the specificity of our HRMA. *JAK2* V617F mutation was screened using allele-specific PCR with an analytic sensitivity of 5% and *MPL* exon 10 mutation using Sanger sequencing as previously described.^9, 10^ TA-cloning was performed using the pGEM-T easy vector system (Promega, Madison, CA, USA) as previously described.^8^ At least 10 clones in each individual were randomly selected for the screening of *CALR* exon 9 alterations by Sanger sequencing. All novel single-nucleotide variant that was only detected once was treated as artifact and were excluded. The SPSS Statistics software (IBM, New York, NY, USA) was used for all calculations. *P*-values<0.05 were considered significant.

Among the 92 ET patients (median age 53 years; 58% females), 59 (64%) patients harbored *JAK2* V617F mutation and one (1%) patient harbored *MPL* W515K mutation. Thirty-two *JAK2*/*MPL*-unmutated ET patients were utilized for the development of our HRMA platform.^8^ Briefly, 22 (68.8%) samples were found to have distinct melting curves from wild type. In 16 of these 22 samples, Sanger sequencing confirmed the presence of six types of *CALR* mutations: five type 1 (p.L367fs*46), six type 2 (p.K385fs*47), one type 3 (p.L367fs*48), two type 34 (p.K385fs*47) and two other types (p.L367fs*43 and p.E369fs*50). The other six samples were wild type by sequencing, and *CALR* type 2 mutations were detected in five of six patients after TA-cloning, indicating the presence of low-allele-burden *CALR* mutants in them. By using our HRMA platform, we identified *CALR* mutations in 21 (22.8% overall and 65.6% in *JAK2*/*MPL*-unmutated) ET patients and this frequency is comparable to other studies.^2, 3, 4^ Eleven (12%) ET patients were negative for *JAK2*, *CALR* and *MPL* mutations. In the 78 samples from healthy adults, two were found with HRMA to have distinct melting curves from wild type. One single-nucleotide polymorphism (rs143880510) and one wild type were found after Sanger sequencing in these two samples. Therefore, our HRMA system has a low false-positive rate of 1.3%.

After screening the 59 *JAK2* V617F-mutated ET patients for *CALR* alterations by HRMA, 16 (27.1%) samples were found to have distinct melting curves from wild type (Figure 1). In 2 of these 16 samples, one *CALR* type 3 mutation (p.L367fs*48) and one single-nucleotide polymorphism (rs143880510) were detected using Sanger sequencing. All the other 14 samples were wild type by sequencing. Interestingly, we detected a high frequency of *CALR* exon 9 alterations in 12 (85.7%) of these 14 patients after TA-cloning (Table 1A). Three patients harbored the classic *CALR* indel mutations: one each of type 2 p.K385fs*47, p.E370fs*60 and p.E371fs*59. Hence, four (6.8%) ET patients had classic *CALR* indel and *JAK2* V617F co-mutations in this cohort. Five patients (8.5%) including the aforementioned patient (P520) with type 2 *CALR* mutation harbored four types of 3-bp inframe deletions all resulted in the deletion of a single amino acid of glutamic acid: two p.E381del and one each of p.E371del, p.E378del and p.E396del (Supplementary Figure 1). Another five patients (8.5%) harbored five types of point mutations: one each of p.E374X, p.E380X, p.K391X, p.E372G and p.E380G. The latter p.E380G has been reported as an single-nucleotide polymorphism but might be a low-allele-burden somatic mutation in this patient because it was only detected after TA-cloning and not by Sanger sequencing on patient's genomic DNA. The remaining two patients were found to have wild-type *CALR* exon 9 after screening for 100 independent clones, and were counted as *CALR* wild type. Overall, various *CALR* exon 9 alterations were detected in 13 (22%) of 59 *JAK2* V617F-mutated ET patients.

We then examined the clinical and molecular correlates in 91 ET patients excluding the one *MPL*-mutated patient (Table 1B). *JAK2*-mutated ET patients with concomitant *CALR* alterations were associated with oldest age (*P*=0.025), higher thrombotic events after diagnosis (*P*=0.048), higher major arterial thrombotic events after diagnosis (*P*=0.022) and more patients being in the high-risk group for thrombohemorrhagic complications (*P*=0.023). Consistent with previous reports, *CALR* mutations were associated with younger age (*P*=0.025), higher platelet count (*P*<0.001) and lower hemoglobin level (*P*=0.016). *JAK2* V617F mutation was associated with leukocytosis (*P*=0.046).

After the discovery of *CALR* mutations, it has been proposed to be mutually exclusive with *JAK2* and *MPL* mutations in MPN. However, *CALR* and *JAK2* V617F co-mutations have been reported in a few MPN cases across different ethnic groups and the frequency is usually below 1%.^7, 11, 12, 13^ In contrast to these reports, we detected a higher frequency of 6.8% *CALR* indel and *JAK2* co-mutations in ET patients. Interestingly, three of these *CALR* mutations were low-allele-burden mutants not detected using Sanger sequencing. Nevertheless, the use of a sensitive HRMA technique has enabled us to detect these low-allele-burden *CALR* mutants in both *JAK2*-mutated and *JAK2*/*MPL*-unmutated ET patients. In addition, we also detected several *CALR* exon 9 point mutations and inframe deletions in *JAK2*-mutated ET patients, but none in our *JAK2*/*MPL*-unmutated ET patients. Recently, point mutations in *CALR* were also reported in follicular lymphoma (E403X and E405Q), PMF (E379D) and chronic neutrophilic leukemia (E398D).^14^ Two rare inframe deletions in *CALR* exon 9 (p.E393_E395del and p.E405del) have been reported in the National Heart, Lung, and Blood Institute Grand Opportunity Exome Sequencing Project with undetermined significance. All the five inframe deletions we detected were 3-bp deletions similar to the latter one. Although the possibility of low-allele-burden germline sequence variations cannot be completely excluded, these 3-bp inframe deletions detected using HRMA were more likely to be low-allele-burden somatic mutations not detected using Sanger sequencing in our patients. Recently, *CALR* point mutations (E381A and D373M) and inframe deletions (E381_A382>A, D397_D400>D, D400_K401>D and E405_V409>V) were also detected in patients with suspected MPN and *JAK2*-mutated MPN in another study albeit with a lower frequency.^15^ These *CALR* alterations were also found to co-occur with *MPL, CSF3R*, *ASXL1* and *ZRSR2*. Currently, the role of these *CALR* point mutations and inframe deletions in the molecular pathogenesis of MPN is not yet clear. Because they frequently co-occurred with mutations involving the JAK-STAT pathway and affected disease phenotype in *JAK2*-mutated ET patients, these non-classic *CALR* mutant proteins are suspected to have a contributory role in the pathogenesis of MPN.^15^ The frequency of these non-classic *CALR* mutations in PMF and other MPN requires further study.

In conclusion, we have detected a high frequency of both classic and non-classic *CALR* exon 9 alterations in *JAK2*-mutated ET patients by HRMA. The presence of *CALR* alterations in *JAK2*-mutated ET defines a specific subgroup of patients requiring careful follow-up and management for their increased risk of thrombotic events. Because our study is limited by small patient number, larger study is warranted to confirm our observation.

## Figures and Tables

**Figure 1:**
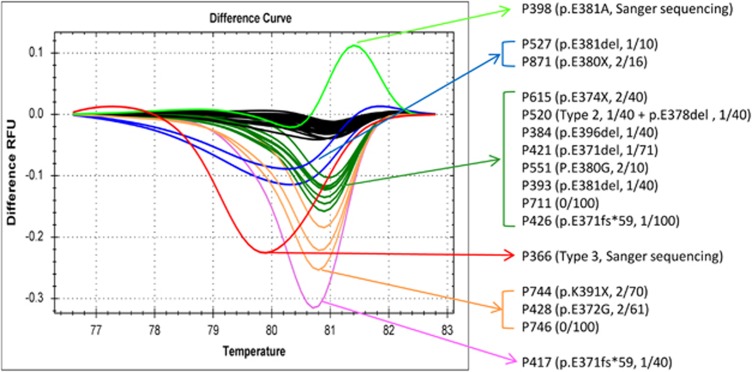
Normalized difference curves of 16 *JAK2* V617F-mutated essential thrombocythemia patient samples showing distinct melting curves from *CALR* exon 9 wild-type samples (black color). Corresponding patient number, genotype and number of positive clone in TA-cloning of each curve is indicated by arrow.

**Table 1A:** *CALR* exon 9 alterations and single-nucleotide polymorphism in 14 *JAK2* V617F-mutated essential thrombocythemia patients detected using high-resolution melting analysis

*Patient*	*CALR mutation*	*Nucleotide change*	*Protein change*	*Amino acid*	*Protein sequence*^a^	*CALR SEQ*	*CALR-TA clone number*^b^	*JAK2 V617F allele burden*^c^
NA	Wild type	NA	NA	417	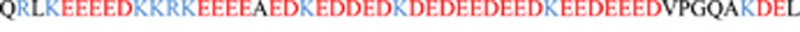	Wild type	NA	NA
P520	Type 2	c.1154_1155insTTGTC	p.K385fs^a^47	430	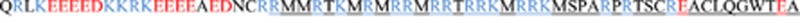	Wild type	1/40	7%
P366	Type 3	c.1095_1140del (Δ46)	p.L367fs^a^48	413	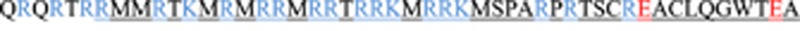	Heterozygous	NA	83%
P426	New	c.1108delG (Δ1)	p.E370fs^a^60	428	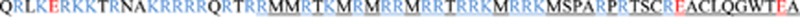	Wild type	1/100	25%
P417	New	c.1111delG (Δ1)	p.E371fs^a^59	428	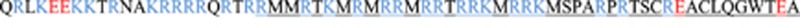	Wild type	1/40	20%
P421	New	c.1110_1112delGGA (Δ3)	p.E371del	416	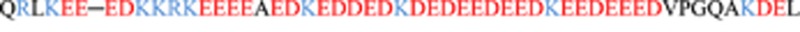	Wild type	1/71	71%
P520	New	c.1132_1134delGAG (Δ3)	p.E378del	416	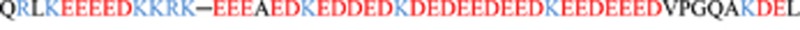	Wild type	1/40	7%
P393	New	c.1142_1144delAGG (Δ3)	p.E381del	416	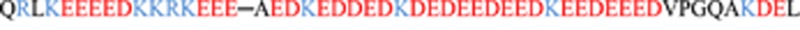	Wild type	1/40	5%
P527	New	c.1142_1144delAGG (Δ3)	p.E381del	416	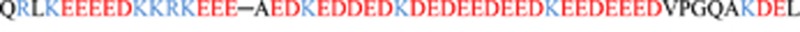	Wild type	1/10	4%
P384	New	c.1188_1190delGGA (Δ3)	p.E396del	416	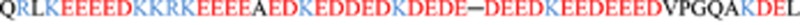	Wild type	1/40	27%
P615	New	c.1120A>T	p.E374X	373	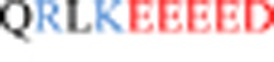	Wild type	2/40	23%
P871	New	c.1138 G>T	p.E380X	379	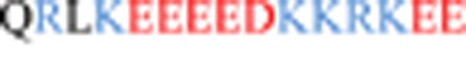	Wild type	2/16	32%
P744	New	c.1171A>T	p.K391X	390	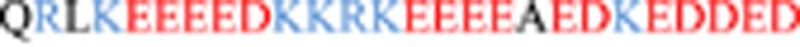	Wild type	2/70	13%
P428	New	c.1115A>G	p.E372G	417	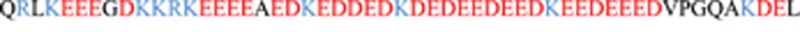	Wild type	2/61	50%
P551	rs201971744	c.1139A>G	p.E380G	417	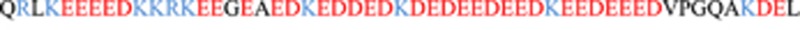	Wild type	2/10	41%
P398	rs143880510	c.1142A>C	p.E381A	417	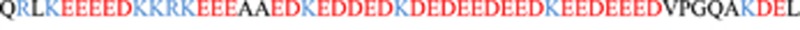	Heterozygous	NA	26%

Abbreviations: NA, not available; PCR, polymerase chain reaction; SEQ, Sanger sequencing.

a Red and blue fonts indicate acidic and basic amino acids, respectively. Underline indicates the same C-terminal sequence changes after +1 base pair reading frameshift.

b TA clones of the *CALR* PCR products amplified from each colony were analyzed using Sanger sequencing. The total number of clones examined and the number of each genotype are listed in the table.

c Based on the relative peak areas of the mutant and wild-type PCR products in Sanger sequencing. All patients were tested positive for *JAK2* V617F mutation using allele-specific PCR.

**Table 1B:** Clinical and laboratory characteristics at diagnosis or referral of 91 essential thrombocythemia patients stratified by mutation profiles

*Variables*	*All (*n=*91)*	*A. JAK2 V617F mutation (*n=*46)*	*B. CALR mutation (*n=*21)*	*C. JAK2-mutated and CALR alterations (*n=*13)*	*D. Triple-negative (*n=*11)*	*A vs B vs C vs D* P *value*	*A vs B vs C* P *value*	*A vs C* P *value*	*B vs C* P *value*
Male/female gender, *n* (%)	39/52 (43/57)	21/25 (46/54)	9/12 (43/57)	5/8 (39/61)	4/7 (36/64)	NS	NS	NS	NS
Age at diagnosis (years), median (range)	53 (22–89)	54.5 (25–89)	47 (22–76)	60 (26–80)	52 (35–79)	0.025	0.012	NS	0.004
Follow-up (years), median (range)	3.7 (0.02–23.1)	3.6 (0.04–23.1)	5,4 (0.5–13.2)	3.8 (0.02–6.1)	3.1 (0.2–10.3)	NS	NS	NS	0.032
History of thrombosis, *n* (%)	19 (20.9)	9 (19.6)	3 (14.3)	5 (38.5)	2 (18.2)	NS	NS	NS	NS
Major thrombosis, *n* (%)	17 (18.7)	8 (17.4)	2 (9.5)	5 (38.5)	2 (18.2)	NS	NS	NS	NS
Thrombosis after diagnosis, *n* (%)	10 (11)	3 (6.5)	2 (9.5)	4 (30.8)	1 (9.1)	NS	0.048	0.036	NS
Major arterial thrombosis after diagnosis, *n* (%)	6 (6.6)	1 (2.2)	1 (4.8)	3 (23.1)	1 (9.1)	NS	0.022	0.03	NS
History of hemorrhage, *n* (%)	25 (27.5)	13 (28.3)	9 (42.9)	2 (15.4)	1 (9.1)	NS	NS	NS	NS
Major hemorrhage, *n* (%)	17 (18.7)	9 (19.6)	6 (28.6)	2 (15.4)	0	NS	NS	NS	NS
High-risk group for thrombohemorrhagic complications^a^, *n* (%)	43 (47.3)	22 (47.8)	6 (28.6)	10 (76.9)	5 (45.5)	NS	0.023	NS	0.012
Hemoglobin (g dl^−1^), median (range)	13.3 (4.5–17.9)	14.0 (4.5–17.9)	12.6 (8.5–15.2)	13.3 (8.8–16.6)	12.8 (9.3–15.2)	0.016	0.016	NS	NS
WBC (x10^3^ μl^−1^), median (range)	10.3 (4.8–29.9)	12.1 (4.8–29.9)	9.2 (4.9–27.9)	11.8 (6.0–24.2)	8.2 (5.3–25.5)	0.046	NS	NS	NS
Platelets (x10^9^ l^−1^), median (range)	936 (335–2834)	942 (335–1496)	1351 (642–2834)	855 (547–1931)	708 (532–1374)	<0.001	<0.001	NS	0.001

Abbreviations: *n*, number; NS, not significant; WBC, white blood cell.

a High-risk group for thrombohemorrhagic complications: Age ⩾60 years and/or a previous history of thrombosis.
