# Supplementary figures and images for: Frequent CALR exon 9 alterations in JAK2 V617F-mutated essential thrombocythemia detected by high-resolution melting analysis
Source: Blood Cancer J. 2015 Mar 20;5(3):e295–. doi: 10.1038/bcj.2015.21 (PMC4382662; doi:10.1038/bcj.2015.21)

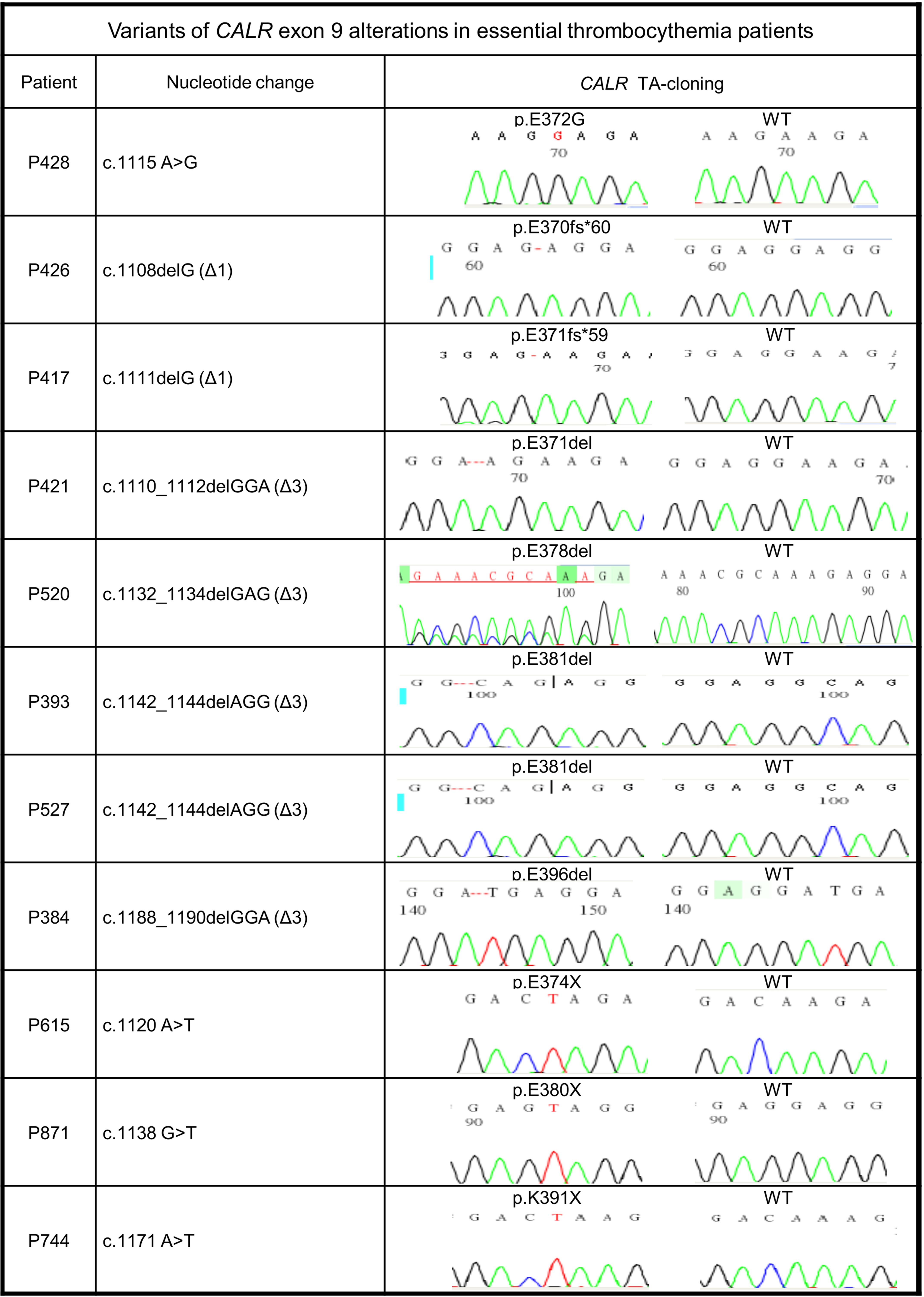

Supplement: Supplementary Figure 1 [file bcj201521x1.tif]
