# Supplementary material for: Frequent CALR exon 9 alterations in JAK2 V617F-mutated essential thrombocythemia detected by high-resolution melting analysis
Source: Blood Cancer J. 2015 Mar 20;5(3):e295–. doi: 10.1038/bcj.2015.21 (PMC4382662; doi:10.1038/bcj.2015.21)
Supplement: Supplementary Figure Legend [file bcj201521x2.doc]

Supplementary Figure 1. Variants of *CALR* exon 9 alterations in essential thrombocythemia patients detected by high-resolution melting analysis followed by TA-cloning. WT, wild-type.
